# Population Pharmacokinetics of Rifampicin in Plasma and Cerebrospinal Fluid in Adults With Tuberculosis Meningitis

**DOI:** 10.1093/infdis/jiaf178

**Published:** 2025-04-29

**Authors:** Noha Abdelgawad, Sean Wasserman, Kamunkhwala Gausi, Angharad Davis, Cari Stek, Lubbe Wiesner, Graeme Meintjes, Robert J Wilkinson, Paolo Denti

**Affiliations:** Division of Clinical Pharmacology, Department of Medicine; Wellcome Discovery Research Platforms for Infection, Centre for Infectious Diseases Research in Africa, Institute of Infectious Disease and Molecular Medicine, University of Cape Town, Observatory, South Africa; Institute for Infection and Immunity, City St George's University of London, London, United Kingdom; Division of Clinical Pharmacology, Department of Medicine; Wellcome Discovery Research Platforms for Infection, Centre for Infectious Diseases Research in Africa, Institute of Infectious Disease and Molecular Medicine, University of Cape Town, Observatory, South Africa; The Francis Crick Institute, London, United Kingdom; Queen Mary and Barts Tuberculosis Centre, Blizard Institute, Faculty of Medicine and Dentistry, Queen Mary University London, United Kingdom; Wellcome Discovery Research Platforms for Infection, Centre for Infectious Diseases Research in Africa, Institute of Infectious Disease and Molecular Medicine, University of Cape Town, Observatory, South Africa; Division of Clinical Pharmacology, Department of Medicine; Wellcome Discovery Research Platforms for Infection, Centre for Infectious Diseases Research in Africa, Institute of Infectious Disease and Molecular Medicine, University of Cape Town, Observatory, South Africa; Department of Medicine, University of Cape Town, Observatory, South Africa; Blizard Institute, Queen Mary University of London, United Kingdom; Wellcome Discovery Research Platforms for Infection, Centre for Infectious Diseases Research in Africa, Institute of Infectious Disease and Molecular Medicine, University of Cape Town, Observatory, South Africa; The Francis Crick Institute, London, United Kingdom; Division of Infectious Diseases and HIV Medicine, Department of Medicine, University of Cape Town, Observatory, South Africa; Department of Infectious Diseases, Imperial College London, United Kingdom; Division of Clinical Pharmacology, Department of Medicine

**Keywords:** cerebrospinal fluid, high-dose rifampicin, modeling and simulation, population pharmacokinetics, tuberculosis meningitis

## Abstract

**Background:**

Several ongoing clinical trials are evaluating high-dose rifampicin (up to 35 mg/kg) for tuberculous meningitis (TBM). However, rifampicin pharmacokinetics at higher doses is not fully characterized, particularly in cerebrospinal fluid (CSF), the site of TBM disease.

**Methods:**

In a randomized controlled trial, adults with HIV-associated TBM were assigned to experimental arms of high-dose rifampicin (oral, 35 mg/kg; intravenous, 20 mg/kg) plus linezolid, with or without aspirin, or a control arm that received the standard of care with 10 mg/kg of oral rifampicin. Rifampicin concentrations, including the unbound fraction, were measured on plasma samples, and CSF was collected on days 3 and 28 of study enrollment. Data were analyzed by nonlinear mixed effects modeling.

**Results:**

In total, 400 plasma and 44 CSF rifampicin concentrations from 48 participants were used for model development. The median (range) age and weight were 39 years (25–78) and 60 kg (30–107). Rifampicin pharmacokinetics was best described by a 2-compartment disposition model with first-order transit oral absorption and elimination via saturable hepatic extraction. Typical clearance values for the standard dose for days 3 and 28 were 33.1 and 41.4 L/h, respectively; high-dose values were 46.1 and 70.2 L/h. The CSF-plasma ratio was approximately 6% and the equilibration half-life was 3.2 hours. Simulated standard-dose rifampicin did not reach CSF concentrations above the critical concentration for *Mycobacterium tuberculosis*.

**Conclusions:**

CSF penetration with standard-dose rifampicin is low. Our findings support continued evaluation of high-dose rifampicin for TBM treatment.

## Teaser

We describe rifampicin pharmacokinetics at high and standard doses (35 and 10 mg/kg) in the plasma and cerebrospinal fluid in adults with tuberculous meningitis/HIV. Cerebrospinal fluid penetration with standard-dose rifampicin is low. Our findings support the ongoing evaluation of high-dose rifampicin for tuberculous meningitis treatment.

Tuberculosis meningitis (TBM) is a severe form of extrapulmonary tuberculosis (TB) involving the central nervous system (CNS). Despite treatment, case fatality is approximately 25% in individuals who are HIV negative and up to 50% in those with HIV, with permanent disability in approximately 20% of survivors [1, 2]. Most deaths from TBM occur early in the illness; therefore, more rapid and effective treatment may improve outcomes [3].

One way to achieve this is to optimize dosing to ensure attainment of therapeutic drug concentrations in the CNS, the site of the disease [2]. Drugs targeted at TBM need to cross several barriers, including the blood-brain barrier and the blood–cerebrospinal fluid barrier, which separate systemic circulation from the CNS. This should occur rapidly and at adequate concentrations for effective antitubercular activity. Disease-related changes in blood-brain barrier permeability and dynamic changes in protein concentrations may importantly influence drug penetration into the CNS [4].

Rifampicin remains the cornerstone of TBM management but is still dosed at 10 mg/kg, the same as for pulmonary TB, whose disease site is in the lungs. Clinical studies of pulmonary TB show a correlation between rifampicin dose and sputum culture conversion [5], suggesting that higher doses may be beneficial to achieve higher site-of-disease exposures and potentially better clinical outcomes. There is evidence that rifampicin doses ≥30 mg/kg are required to achieve adequate intralesional concentrations in TBM [6] and that CSF concentrations increase with higher doses [7]. This has led to several trials investigating higher-dose rifampicin for TBM: clinical outcomes of this approach have been variable, and there remains uncertainty around the dose selection that optimizes CSF exposure.

Orally administered rifampicin is well absorbed, with >86% absolute bioavailability [8]. It exhibits nonlinear elimination with a saturable first-pass effect at higher doses [9, 10], and it induces many enzymes and transporters via activation of the pregnane X receptor, including those involved in its clearance. For this reason, rifampicin clearance is expected to double after about 2 weeks of administration [11]. It is highly protein bound (approximately 80%), with poor penetration in CNS tissues [12]. Rifampicin plasma pharmacokinetics has been extensively described among pulmonary TB populations, but limited data are available on its CSF pharmacokinetics, especially among patients with TBM where disease effects may influence penetration and equilibration into this compartment. We therefore characterized rifampicin pharmacokinetics in plasma and CSF in adults with HIV-associated TBM following the administration of high-dose rifampicin, either orally (35 mg/kg) or intravenously (20 mg/kg), and standard-dose rifampicin orally (10 mg/kg).

## METHODS

### Parent Study and Interventions

This was a pharmacokinetic substudy of LASER-TBM, a phase 2A trial investigating the safety and tolerability of intensified antibiotic therapy in adults with HIV-associated TBM. Participants were enrolled within 5 days of anti-TB treatment initiation from 4 hospitals in South Africa and randomly assigned to either a control group or 1 of 2 experimental regimens. The control group received the standard-of-care TBM regimen according to World Health Organization weight bands (rifampicin, 10 mg/kg; isoniazid, 5 mg/kg; pyrazinamide, 25 mg/kg; ethambutol, 15 mg/kg), which was administered as fixed-dose combination (FDC) tablets. Individuals allocated to experimental arms received additional rifampicin plus daily oral linezolid (1200 mg) with or without aspirin. They underwent a second randomization to receive either high-dose oral (35 mg/kg) or intravenous (IV; 20 mg/kg) rifampicin for the first 3 days of treatment; after day 3, all participants in the experimental arms continued oral high-dose rifampicin (35 mg/kg) daily until the end of the study. High-dose oral rifampicin was administered as FDC tablets topped with individual rifampicin tablets according to bespoke weight bands designed to balance exposures across weight groups [13]. Those in the IV rifampicin group received the full rifampicin dose (20 mg/kg) as a 1-hour infusion. All participants received adjunctive corticosteroids. More details about LASER-TBM can be found in the publication by Davis et al [14].

The study was approved by the University of Cape Town Human Research Ethics Committee (reference 293/2018), Walter Sisulu University (reference 012/2019), and the South African Health Products Regulatory Authority (reference 20180622). The trial was registered on clinicaltrials.gov (NCT03927313). Informed consent was obtained from all participants or their proxies.

### Pharmacokinetic Sampling

All trial participants underwent pharmacokinetic sampling on days 3 (visit 1) and 28 (visit 2) ±2 days after study enrollment. Plasma samples were collected predose and 0.5, 1, 2, 3, 6, 8 to 10, and 24 hours postdose on day 3 and predose and 2 and 4 hours postdose on day 28. One lumbar CSF sample was collected at each sampling visit, with sampling time randomized to intervals of 1 to 3, 3 to 6, 6 to 10, and 24 hours postdose. Immediately following collection, samples were processed on-site and stored at −80 °C. Total rifampicin concentrations (protein bound and unbound) were quantified in all collected plasma and CSF samples, and free plasma rifampicin concentrations (unbound) were measured in a subset of participants. Drug quantification was performed by a validated liquid chromatography–tandem mass spectrometry assay developed at the Division of Clinical Pharmacology, University of Cape Town. Additional details regarding the assay method are presented in the supplementary material. The unbound fraction (fu) of plasma rifampicin was estimated by Deming regression to regress measured free concentrations against total concentrations with an intercept of zero [15, 16]. Participant characteristics, clinical information, and blood chemistry were obtained on each visit. Total protein, albumin, and glucose were measured in CSF samples.

### Pharmacokinetic Modeling

Population pharmacokinetic modeling was used to describe total rifampicin plasma and CSF concentrations. The model was developed stepwise: first, we used the IV dosing plasma concentrations to test different disposition models; second, we included the oral dosing plasma concentrations; finally, we added the CSF concentrations. For the CSF “effect” compartment, we estimated a pseudo-partition coefficient (PPC; ie, CSF to plasma drug ratio) and an equilibration half-life (*T*_1/2eq_; ie, the delay in equilibration between the plasma and CSF, as described in the supplementary material). All plasma and CSF parameters were estimated simultaneously. A previously published model by Chirehwa et al [11] was used as a starting point to develop the plasma pharmacokinetic model. We compared saturable hepatic elimination and first-pass effect vs linear clearance. We also tested different approaches to describe clearance autoinduction. These included an exponential model with clearance increasing as a function of days on rifampicin treatment [11], an enzyme turnover model [10], and estimation of separate values for typical intrinsic clearance and the fraction unbound (CL_int,max_·f_u_) for each visit. Allometric scaling was applied for all disposition parameters via the fixed power exponents of 0.75 for clearance parameters and 1 for volume parameters [17]. Either total body weight or fat-free mass (FFM; calculated by the formula from Janmahasatian et al [18]) was tested as a body size descriptor. Lag time and transit compartments were tested to capture the delay in absorption. We tested the inclusion of between-subject and between-visit variabilities for the disposition parameters and bioavailability and between-occasion variability for the absorption parameters. An occasion was defined as any dosing event with at least 1 observation postdose, and a visit refers to a predose sample, dosing, and postdose observations on a visit to the clinical site.

The influence of potential covariates on plasma pharmacokinetic parameters was tested following the development of the structural model. Covariates included the effect of rifampicin dose, duration since start of treatment, and study arm. We also tested whether FDC tablets and top-up individual tablets had significantly different bioavailability, as previously reported [19, 20]. The effects of various laboratory and clinical covariates were tested on PPC, including CSF protein, CSF albumin, CSF glucose, polymorphonuclear cells, lymphocytes, and the Glasgow Coma Scale. Additional details for the modeling methods and imputation of missing covariates are provided in the supplementary material. Covariate selection was based on the drop in objective function value (dOFV) and physiologic plausibility.

### Simulations

The final model was used to predict the area under the concentration-time curve from time 0 to 24 hours postdose (AUC_0–24h_) and the concentration at 24 hours postdose for the available concentration-time profiles. We also predicted concentration-time profiles in plasma and CSF following standard-dose (10 mg/kg) and high-dose (35 mg/kg) rifampicin for a typical participant in this cohort. The critical concentration (CC) referenced in the World Health Organization technical report on CCs for drug susceptibility testing of isoniazid and the rifamycins (rifampicin, rifabutin, and rifapentine) [21] was used as a reference value. Minimum inhibitory concentration tests were not performed on *Mycobacterium tuberculosis* isolates from the patients of this study.

## RESULTS

### Study Data

Forty-nine participants underwent pharmacokinetic sampling on day 3, and 34 participants were sampled on day 28, providing 411 plasma samples (56 below limit of quantitation) and 46 CSF samples (13 below limit of quantitation) for model development. We excluded rifampicin concentrations from 1 participant, who had observations only for the first visit due to dislocation of the IV catheter and tissue extravasation of the drug.

Baseline clinical characteristics are summarized in Table 1. The median (range) age, weight, and FFM were 39 years (25–78), 60 kg (30–107), and 45 kg (30–59), respectively. The median (range) duration since the start of rifampicin-based TB treatment was 5 days (1–8) on the first visit and 30 days (26–38) on the second.

**Table 1.** Participant Characteristics

	Median (Range) or No. (%)	
	Visit Day 3 (n = 49)	Visit Day 28 (n = 34)
Males	27 (55.1)	20 (58.8)
Age, y	39 (25–78)	39 (25–57)
Weight, kg	59.5 (30–107.2)	61.7 (37.4–105.1)
Height, m^a^	1.60 (1.48–1.80)	1.60 (1.49–1.80)
Fat-free mass, kg^b^	45.2 (30.3–59.4)	45.5 (32.4–60)
Days taking rifampicin until PK visit date^c^	4 (0–7)	30 (26–38)
CSF		
Total protein, g/L^d^	1.16 (0.2–55)	1.21 (0.2–55)
Albumin, mg/L^e^	387 (46–7601)	373 (46.0–1269)
Glucose, mmol/L^d^	3.05 (0.05–5.9)	2.8 (0.3–5.9)
Antiretroviral therapy		
Previous	14 (28.6)	10 (29.4)
Naive	20 (40.8)	14 (41.2)
Undergoing	15 (30.6)	10 (29.4)

Abbreviation: CSF, cerebrospinal fluid; PK, pharmacokinetics.

^a^Heights were missing for 29 and 19 participants on days 3 and 28, respectively. The missing heights were imputed by sex and weight according to the details provided in the supplementary file. The median (range) values reported here are for the nonmissing values (ie, they do not include the imputed values).

^b^Fat-free mass was calculated by sex, weight, and height according to the formula from Janmahasatian et al [18]. The median (range) values reported here are for the nonmissing values (ie, they do not include the imputed values).

^c^This refers to the total number of days since the start of treatment, which was approximately 1 to 3 days before the investigational product start date until the PK visit date. All participants were assumed to be taking standard-dose rifampicin (10 mg/kg) when starting treatment and before study enrollment.

^d^CSF total protein and glucose values were missing for 17 and 8 participants on days 3 and 28, respectively.

^e^CSF albumin values were missing for 23 and 13 participants on days 3 and 28, respectively.

### Pharmacokinetic Modeling

**Figure 1.**
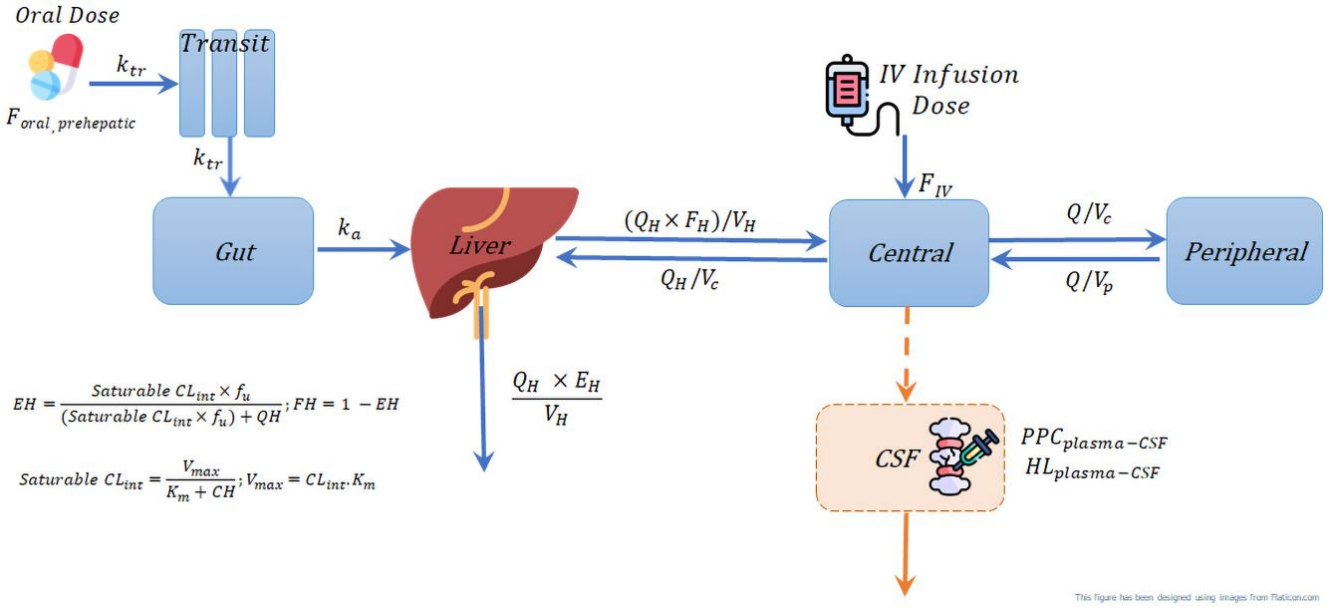
Illustration of the final structural model (from left to right): *F*_oral,prehepatic_ is the prehepatic oral bioavailability. *k_tr_* is the first-order rate constant for drug passage through transit compartments. *k_a_* is the first-order absorption rate constant. *Q_H_* is the hepatic blood flow. *F_H_* is the hepatic bioavailability. *E_H_* is the hepatic extraction. *V_H_* is the hepatic volume of distribution. *V_c_* is the central volume of distribution. *F_IV_* is the absolute intravenous bioavailability. *V_p_* is the peripheral volume of distribution. *Q* is the intercompartmental clearance. *K_m_* is the Michaelis-Menten constant. *V_max_* is the maximum rate of elimination. *C_H_* is the hepatic drug concentration. *CL_int_* is the intrinsic clearance. PPC_plasma-CSF_ is the pseudo-partition coefficient, which represents the ratio of drug in CSF to plasma, and HL_plasma-CSF_ is the equilibration half-life between plasma and CSF, which describes how soon the change in plasma is reflected in the CSF. CSF, cerebrospinal fluid; IV, intravenous.

Plasma pharmacokinetics of rifampicin concentrations was best described by a 2-compartment disposition model vs a 1-compartment disposition (dOFV, −10.9; *P* = .004), with first-order absorption preceded by a chain of transit compartments and elimination with saturable hepatic extraction. A depiction of the structural model is shown in Figure 1. Robust estimation of the *K_m_* was challenging; therefore, we used a prior value from the model by Chirehwa et al [11] to guide its estimation. The model fit improved significantly upon including clearance saturation vs linear clearance (dOFV, 10.6; *P* < .005).

Considering that the bioavailability of the IV dose was 100%, oral prehepatic bioavailability was estimated at 93.4% before first-pass hepatic extraction. Clearance was higher in participants receiving larger doses of rifampicin and at visit 2 as compared with visit 1: attempts to describe this by the exponential change over time suggested by Chirehwa et al [11] or by an enzyme turnover model similar to that by Svensson et al [10] (also with prior values to help guide parameter estimation) did not result in robust parameter estimation, as the models did not converge properly. Therefore, clearance was estimated by separate CL_int,max_·f_u_ values for each visit. The typical CL_int,max_·f_u_ values for the standard dose were 33.1 L/h for visit 1 and 41.4 L/h for visit 2, while the high-dose values were 46.1 L/h for visit 1 and 70.2 L/h for visit 2. Allometry with FFM resulted in better model fit as compared with body weight (dOFV, 33.1 points [*P* < .0001] for FFM vs 13.7 points [*P* < .001] for body weight). We did not find a statistically significant difference in bioavailability for the FDC and the individual top-up tablets or for biomarkers such as creatinine, aspartate aminotransferase, and alanine aminotransferase.

CSF equilibration half-life and the PPC were estimated to be 3.1 hours and 5.3%, respectively. None of the covariates tested resulted in a statistically significant effect on the PPC or the equilibration half-life. All parameter estimates are presented in Table 2, and the visual predictive check for the plasma and CSF observations is displayed in Supplementary Figure 1, showing good agreement between documented concentrations and model predictions.

**Table 2.** Final Population Pharmacokinetic Parameter Estimates for Rifampicin in Plasma and CSF

Parameter^a^	Estimate (95% CI)^b^
CL_int,max_·f_u_, L/h^c^	
Standard dose: visit 1	33.1 (25.2–42.9)
Standard dose: visit 2	41.4 (28.0–58.3)
High dose: visit 1	46.1 (34.2–62.3)
High dose: visit 2	70.2 (51.0–95.5)
Michaelis-Menten constant, *K_m_*, mg/L^d^	2.97 (2.01–4.56)
Central volume of distribution, *V*, L^c^	27.3 (22.8–34.0)
Bioavailability	
Prehepatic oral^e^	0.934 (0.852–0.991)
Intravenous, *F*	1 fixed
Peripheral volume of distribution, *V_p_*, L	31.5 (25.3–37.1)
Intercompartmental flow, *Q*, L/h	11.0 (7.45–14.4)
Absorption rate constant, *k_a_*, h^−1^	0.486 (0.365–0.638)
Mean transit time, h	0.634 (0.467–0.791)
No. of absorption transit compartments^f^	19 fixed
Equilibration half-life to CSF, HL_plasma-CSF_, h	3.20 (2.06–4.93)
Pseudo-partition coefficient to CSF, PPC_plasma-CSF_	0.0593 (0.0544–0.0672)
Between-subject variability, %	
CL_int,max_·f_u_	25.3 (24.1–26.6)
Central volume	17.2 (14.8–18.5)
Infusion duration^g^	17.0 (14.9–18.6)
Between-occasion variability, %	
* F*_oral,prehepatic_	18.2 (16.7 –20.1)
*k_a_*	78.1 (55.8–95.6)
Mean transit time	111 (87.7–137)
Error: plasma	
Proportional, %	25.2 (22.3–29.7)
Additive, mg/L ^h^	0.0234
Error: CSF	
Proportional, %	98.4 (91.8–99.9)
Additive, µg/mL^i^	2.31 (1.77–2.93)

For definitions of variables, see Figure 1.

Abbreviations: CSF, cerebrospinal fluid; fu, fraction unbound.

^a^Hepatic volume of distribution, hepatic intercompartmental clearance, and fraction unbound were fixed to 1 L, 90 L/h, and 0.2, respectively.

^b^95% CIs were computed with sampling importance resampling.

^c^All disposition parameters were allometrically scaled by fat-free mass. The values reported here refer to the typical participant with a median fat-free mass of 45 kg.

^d^ *K_m_* was estimated by a prior value from Chirehwa et al [11] of 3.35 mg/L with 30% uncertainty.

^e^This refers to the oral bioavailability from the gastrointestinal tract before hepatic extraction.

^f^Number of transit compartments was estimated in earlier runs and fixed in later runs for model stability.

^g^The infusion duration is 1 hour according to the protocol. Between-subject variability was included to account for error in infusion rate duration or time.

^h^The estimate of the additive component of the error was not significantly different from its lower boundary of 20% of the lower limit of quantification (0.117 mg/L), so it was fixed to this value.

^i^The lower boundary of the additive error was fixed to 50% of the lower limit of quantification (0.005 mg/L).

Plasma protein binding was calculated as 82.8%, and there was no evidence of nonlinearities in binding at higher rifampicin concentrations (Supplementary Figure 2).

### Simulations

**Figure 2.**
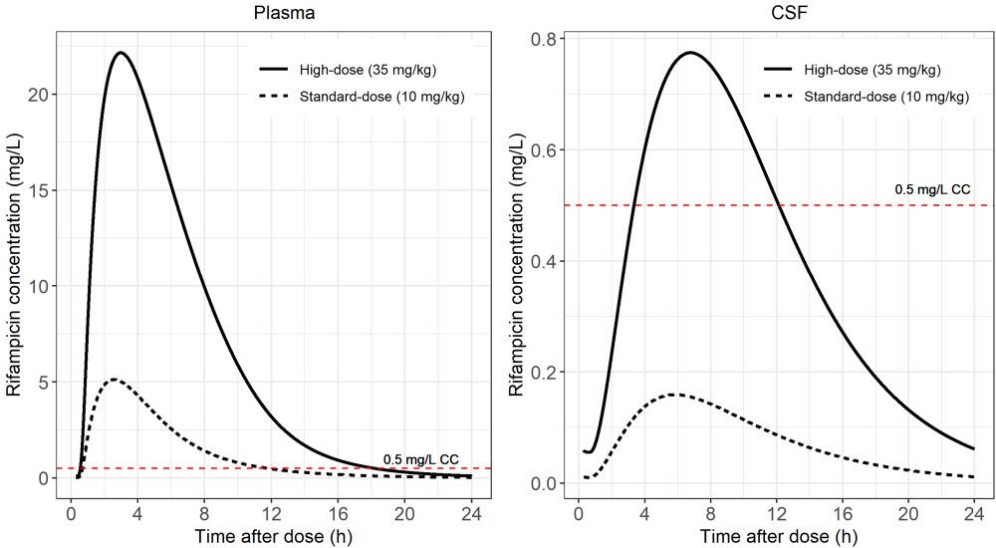
Simulated typical concentration-time profiles for plasma and cerebrospinal fluid (CSF) for oral daily doses at steady state: high (35 mg/kg) and standard (10 mg/kg). The solid and dashed lines represent the high and standard doses, respectively. The horizontal dotted line shows the critical concentration (CC) value of rifampicin for *Mycobacterium tuberculosis* (0.5 mg/L) [21].

**Figure 3.**
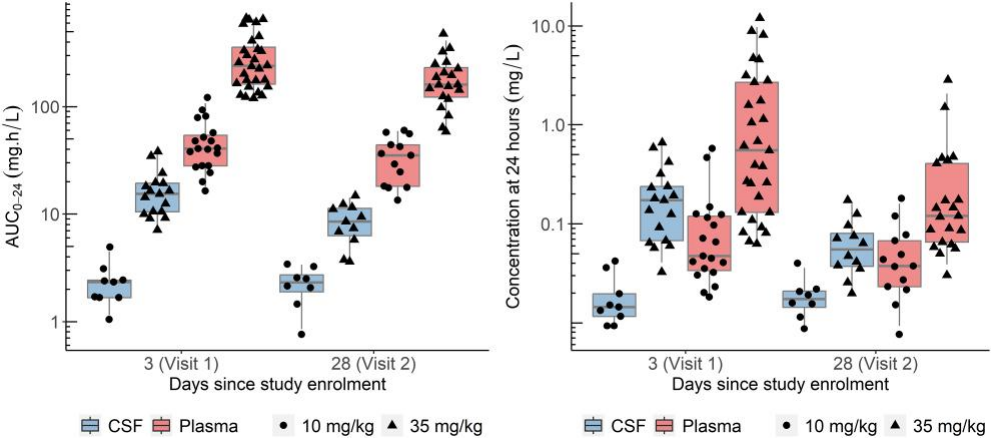
Box and whisker plots show the secondary model-derived exposure parameters: area under concentration curve for 24 hours (AUC_0–24_) and concentration at 24 hours postdose stratified by dose group. The dots represent individual values, and the whiskers are the 2.5th and 97.5th percentiles. Line, median; box, IQR. CSF, cerebrospinal fluid.

The simulated standard-dose CSF profile did not reach concentrations above the rifampicin CC of 0.5 mg/L [21], whereas the high dose achieved concentrations above this CC level (Figure 2). Model-derived individual steady-state AUC_0–24h_ and trough concentrations are summarized in Table 3 and depicted in Figure 3.

**Table 3.** Model-Derived Rifampicin Area Under the Curve for 24 Hours and Concentrations at 24 Hours Postdose

	High Dose, 35 mg/kg				Standard Dose, 10 mg/kg			
	Day 3		Day 28		Day 3		Day 28	
	Median (Range)	No.	Median (Range)	No.	Median (Range)	No.	Median (Range)	No.
AUC_0–24_, mg·h/L								
Plasma	239 (120–668)	22	160 (58.4–477)	20	40.5 (16.4–122)	19	35.1 (13.4–59.8)	13
CSF	15.4 (7.13–38.1)	17	8.50 (3.61–14.8)	11	2.33 (1.05–4.93)	9	2.32 (0.754–3.41)	8
C_24_, mg/L								
Plasma	0.551 (0.0628–12.0)	23	0.120 (0.0303–2.85)	21	0.0473 (0.0182–0.574)	19	0.0374 (0.00758–0.181)	13
CSF	0.173 (0.0327–0.658)	17	0.0556 (0.0199–0.173)	12	0.0144 (0.00929–0.0421)	9	0.0175 (0.00870–0.0399)	8

Abbreviations: AUC_0–24h_, area under the concentration-time curve from time 0 to 24 hours postdose; C_24h_, concentration at 24 hours postdose; CSF, cerebrospinal fluid.

## DISCUSSION

We characterized the pharmacokinetics of rifampicin in plasma and CSF among South African adults with HIV-associated TBM. Our population pharmacokinetic model was based on data following high (35 mg/kg) and standard (10 mg/kg) dosing, as well as oral vs IV administration. The CSF partition coefficient of rifampicin, a measure of drug penetration, was approximately 6%, and the equilibration half-life, indicative of the delay between the plasma and CSF concentrations, was approximately 3 hours. Rifampicin plasma clearance increased over time and was relatively lower for the standard dose than the high dose.

Previous rifampicin CSF models for TBM have been developed through studies in children and adults receiving doses between 15 and 20 mg/kg [22–25]. Their estimates ranged from 4.60% to 8.07% for PPC and between 2.07 and 5.78 hours for equilibration half-life, in line with our findings. An exception is a study by Panjasawatwong et al [24], who reported a PPC of 17% in children with TBM administered rifampicin at 10 mg/kg. While the plasma and CSF concentrations were similar to the standard-dose arm in our study, a different modeling approach was used to estimate CSF penetration. The CSF was modeled as a “real” distribution compartment—with volume fixed for each participant by an empirical formula according to age—and the presence of mass transfer to and from the central compartment. In our analysis, we circumvented the need to estimate CSF volume by modeling CSF as an effect compartment, assuming no volume of distribution for the CSF compartment with no or negligible transfer to and from the plasma.

Similar to Savic et al [22] and Abdelgawad et al [25], we did not find a significant effect from CSF albumin or total protein concentrations, a marker of meningeal inflammation, on rifampicin partitioning into CSF. This is in contrast to studies that have demonstrated a positive correlation between rifampicin CSF penetration and increasing CSF protein levels [23, 24], which could be explained by increased permeability of the protective barriers in TBM, leading to higher concentrations of protein and total drug in the CSF. Another reason could be that increased CSF protein production from local inflammation leads to changes in CSF drug-binding kinetics and higher total drug levels. Measuring free (unbound) drug concentrations in the CSF could provide a clearer understanding of the relationship between CSF proteins and drug concentrations. The lack of an association between CSF protein and drug exposure seen in our study could be due to the small sample size and the narrow range of CSF protein values in our cohort.

Other findings aligned with previous reports. With data obtained from IV administration, we estimated prehepatic oral bioavailability at 93.4%, which aligns with the previously reported absolute bioavailability of >86% [8], although our estimate refers to the fraction of drug available before hepatic extraction. Use of IV data enabled characterization of a 2-compartment disposition model, previously described for rifampicin when IV data were available [7]. The degree of plasma protein binding noted in our study, 82.8%, agrees with the literature for patients with TB [26]. We did not see an increase in unbound fraction with higher plasma concentrations, which would have indicated saturation of plasma protein binding. We did see relatively constant protein binding over the observed concentration range, suggesting that plasma protein binding does not become saturated with higher rifampicin exposures. Lack of saturation up to rifampicin doses of 35 mg/kg was also noted in a study involving patients with pulmonary TB receiving rifampicin [27]. Since only the unbound nonionized fraction can cross membrane barriers and diffuse freely into tissues, this indicates that about 34% of free rifampicin crosses into the CSF.

Simulations based on our model predict that the standard 10-mg/kg dose of rifampicin achieves CSF concentrations far below the CC for *M tuberculosis* for most patients with TBM. This is overcome with the 35-mg/kg dose, supporting ongoing clinical evaluation of high-dose rifampicin for TBM. However, no CSF target concentrations have been established for rifampicin efficacy in TBM, and the CC may be an inappropriate pharmacodynamic measure because it is determined under in vitro conditions that differ substantially from those in the CSF [21].

Our analysis had some limitations. In our model, we were unable to characterize clearance autoinduction semimechanistically using an enzyme turnover model or an exponential maturation model. The reason is that the study participants had started rifampicin treatment approximately a week before the first plasma samples were collected and observations from the uninduced state (first day of treatment) were unavailable. Additionally, there was uncertainty regarding the actual dose and duration of treatment before study enrollment. Also, plasma sampling on the second visit was done only up to 4 hours, thus affecting the estimation of CL_int,max_·f_u_ for the second visit. Furthermore, only 1 CSF sample could be obtained per visit due to the invasive nature of lumbar CSF sampling, with high variability in the CSF observations. This makes it difficult to distinguish between-subject variability from random variability (eg, error due to sample assays), requiring a large proportional error for CSF observations.

In summary, we successfully developed a population pharmacokinetic model for rifampicin in plasma and CSF for adults with HIV-associated TBM. This model provides a tool to identify rifampicin dosing strategies to optimize TBM treatment once exposure targets have been defined. Our major finding was that rifampicin has poor CSF penetration, providing a rationale for ongoing evaluation of high-dose rifampicin and, potentially, novel rifamycin-free regimens to improve treatment outcomes in TBM.

## Supplementary Material

jiaf178_Supplementary_Data

## Supplementary Data

Supplementary materials are available at *The Journal of Infectious Diseases* online (http://jid.oxfordjournals.org/). Supplementary materials consist of data provided by the author that are published to benefit the reader. The posted materials are not copyedited. The contents of all supplementary data are the sole responsibility of the authors. Questions or messages regarding errors should be addressed to the author.

## Notes

***Acknowledgments.*** Computations were performed via facilities provided by the University of Cape Town's ICTS High Performance Computing team: https://ucthpc.uct.ac.za/. Study data were collected and managed in REDCap tools hosted at the University of Cape Town. REDCap is a secure web-based software platform designed to support data capture for research studies [28, 29].

***Data availability.*** The data supporting the findings of this study are available from the corresponding author upon reasonable request.

***Open access.*** For the purpose of open access, the authors have applied for a CC-BY public copyright license to any author accepted manuscript version arising from this submission.

***Financial support.*** This work was supported by the National Institutes of Health (K43TW011421 and U01AI170426) and the Bill & Melinda Gates Foundation (INV 052110) to S. W.; a Wellcome Trust PhD Programme for Clinicians Fellowship (award 175479) to A. D.; the Wellcome Trust (214321/Z/18/Z and 203135/Z/16/Z) and the South African Research Chairs Initiative of the Department of Science and Technology and National Research Foundation of South Africa (grant 64787) to G. M.; the Francis Crick Institute, which is funded by Wellcome Trust (CC2112), Cancer Research UK (CC2112), and UK Research and Innovation (CC2112) to R. J. W.; the National Institutes of Health (R01145436), Meningitis Now, and the NIHR Biomedical Research Centre of Imperial College NHS Trust to R. J. W.; the AIDS Clinical Trial Group; the National Institute of Allergy and Infectious Diseases of the National Institutes of Health (awards UM1 AI068634, AI068636, and AI106701; U01 AI068632); the Infant Maternal Pediatric Adolescent AIDS Clinical Trials Group; the Eunice Kennedy Shriver National Institute of Child Health and Human Development; and the National Institute of Mental Health (grant AI068632) to L. W.

## References

[1] Dodd PJ, Osman M, Cresswell FV, et al The global burden of tuberculous meningitis in adults: a modelling study. PLOS Glob Public Heal 2021; 1:e0000069. 10.1371/journal.pgph.0000069 PMC10021871 36962116

[2] Wasserman S, Davis A, Wilkinson RJ, Meintjes G. Key considerations in the pharmacotherapy of tuberculous meningitis. Expert Opin Pharmacother 2019; 20:1791–5. 31305179 10.1080/14656566.2019.1638912 PMC6763362

[3] Jaipuriar RS, Garg RK, Rizvi I, et al Early mortality among immunocompetent patients of tuberculous meningitis: a prospective study. Am J Trop Med Hyg 2019; 101:357–61. 31237232 10.4269/ajtmh.19-0098 PMC6685555

[4] van Toorn R, Schaaf HS, Laubscher JA, van Elsland SL, Donald PR, Schoeman JF. Short intensified treatment in children with drug-susceptible tuberculous meningitis. Pediatr Infect Dis J 2014; 33:248–52. 24168978 10.1097/INF.0000000000000065

[5] Sekaggya-Wiltshire C, Von Braun A, Lamorde M, et al Delayed sputum culture conversion in tuberculosis–human immunodeficiency virus–coinfected patients with low isoniazid and rifampicin concentrations. Clin Infect Dis 2018; 67:708–16. 29514175 10.1093/cid/ciy179 PMC6094003

[6] Tucker EW, Guglieri-Lopez B, Ordonez AA, et al Noninvasive 11C-rifampin positron emission tomography reveals drug biodistribution in tuberculous meningitis. Sci Transl Med 2018; 10:1–12. 10.1126/scitranslmed.aau0965 PMC6360528 30518610

[7] Cresswell FV, Meya DB, Kagimu E, et al High-dose oral and intravenous rifampicin for the treatment of tuberculous meningitis in predominantly human immunodeficiency virus (HIV)–positive Ugandan adults: a phase II open-label randomized controlled trial. Clin Infect Dis 2021; 73:876–84. 33693537 10.1093/cid/ciab162 PMC8423465

[8] Mariappan TT, Singh S, Pandey R, Khuller GK. Determination of absolute bioavailability of rifampicin by varying the mode of intravenous administration and the time of sampling. Clin Res Regul Aff 2005; 22:119–28.

[9] Boeree MJ, Diacon AH, Dawson R, et al A dose-ranging trial to optimize the dose of rifampin in the treatment of tuberculosis. Am J Respir Crit Care Med 2015; 191:1058–65. 25654354 10.1164/rccm.201407-1264OC

[10] Svensson RJ, Aarnoutse RE, Diacon AH, et al A population pharmacokinetic model incorporating saturable pharmacokinetics and autoinduction for high rifampicin doses. Clin Pharmacol Ther 2018; 103:674–83. 28653479 10.1002/cpt.778 PMC5888114

[11] Chirehwa MT, Rustomjee R, Mthiyane T, et al Model-based evaluation of higher doses of rifampin using a semimechanistic model incorporating autoinduction and saturation of hepatic extraction. Antimicrob Agents Chemother 2015; 60:487–94. 26552972 10.1128/AAC.01830-15 PMC4704145

[12] Acocella G . Clinical pharmacokinetics of rifampicin. Clin Pharmacokinet 1978; 3:108–27. 346286 10.2165/00003088-197803020-00002

[13] Wasserman S, Davis A, Stek C, et al Plasma pharmacokinetics of high-dose oral versus intravenous rifampicin in patients with tuberculous meningitis: a randomized controlled trial. Antimicrob Agents Chemother 2021; 65:e0014021. 33972248 10.1128/AAC.00140-21 PMC7611291

[14] Davis AG, Wasserman S, Stek C, et al A phase 2A trial of the safety and tolerability of increased dose rifampicin and adjunctive linezolid, with or without aspirin, for human immunodeficiency virus–associated tuberculous meningitis: the LASER-TBM trial. Clin Infect Dis 2023; 76:1412–22. 36482216 10.1093/cid/ciac932 PMC10110270

[15] Deming WE . Statistical adjustment of data. Oxford, England: Wiley, 1943.

[16] Linnet K . Performance of Deming regression analysis in case of misspecified analytical error ratio in method comparison studies. Clin Chem 1998; 44:1024–31. 9590376

[17] Anderson BJ, Holford NHG. Mechanism-based concepts of size and maturity in pharmacokinetics. Annu Rev Pharmacol Toxicol 2008; 48:303–32. 17914927 10.1146/annurev.pharmtox.48.113006.094708

[18] Janmahasatian S, Duffull SB, Ash S, Ward LC, Byrne NM, Green B. Quantification of lean bodyweight. Clin Pharmacokinet 2005; 44:1051–65. 16176118 10.2165/00003088-200544100-00004

[19] Kengo A, Nabisere R, Gausi K, et al Dolutegravir pharmacokinetics in Ugandan patients with TB and HIV receiving standard- versus high-dose rifampicin. Antimicrob Agents Chemother 2023; 67:e00430-23. 37850738 10.1128/aac.00430-23 PMC10648962

[20] Court R, Chirehwa MT, Wiesner L, et al Quality assurance of rifampicin-containing fixed-drug combinations in South Africa: dosing implications. Int J Tuberc Lung Dis 2018; 22:537–43. 29663959 10.5588/ijtld.17.0697 PMC5905389

[21] World Health Organization . Technical report on critical concentrations for drug susceptibility testing of isoniazid and the rifamycins (rifampicin, rifabutin and rifapentine). Geneva: World Health Organization; 2021. Licence: CC BY-NC-SA 3.0 IGO. Available at: https://www.who.int/publications/i/item/technical-report-on-critical-concentrations-for-drugsusceptibility-testing-of-isoniazid-and-therifamycins-(rifampicin-rifabutin-and-rifapentine).

[22] Savic R, Ruslami R, Hibma J, et al Pediatric tuberculous meningitis: model-based approach to determining optimal doses of the anti-tuberculosis drugs rifampin and levofloxacin for children. Clin Pharmacol Ther 2015; 98:622–9. 26260983 10.1002/cpt.202 PMC4888594

[23] Svensson EM, Dian S, Te Brake L, et al Model-based meta-analysis of rifampicin exposure and mortality in Indonesian tuberculous meningitis trials. Clin Infect Dis 2020; 71:1817–23. 31665299 10.1093/cid/ciz1071 PMC7643733

[24] Panjasawatwong N, Wattanakul T, Hoglund RM, et al Population pharmacokinetic properties of antituberculosis drugs in Vietnamese children with tuberculous meningitis. Antimicrob Agents Chemother 2020; 65:e00487-20. 33139294 10.1128/AAC.00487-20 PMC7927832

[25] Abdelgawad N, Tshavhungwe M, Rohlwink U, et al Population pharmacokinetic analysis of rifampicin in plasma, cerebrospinal fluid, and brain extracellular fluid in South African children with tuberculous meningitis. Antimicrob Agents Chemother 2023; 67:e0147422. 36815838 10.1128/aac.01474-22 PMC10019224

[26] Boman G, Ringberger VA. Binding of rifampicin by human plasma proteins. Eur J Clin Pharmacol 1974; 7:369–73. 4138537 10.1007/BF00558209

[27] Litjens CHC, Aarnoutse RE, van Ewijk-Beneken Kolmer EWJ, et al Protein binding of rifampicin is not saturated when using high-dose rifampicin. J Antimicrob Chemother 2019; 74:986–90. 30597025 10.1093/jac/dky527

[28] Harris PA, Taylor R, Thielke R, Payne J, Gonzalez N, Conde JG. Research electronic data capture (REDCap)—a metadata-driven methodology and workflow process for providing translational research informatics support. J Biomed Inform 2009; 42:377–81. 18929686 10.1016/j.jbi.2008.08.010 PMC2700030

[29] Harris PA, Taylor R, Minor BL, et al The REDCap Consortium: building an international community of software platform partners. J Biomed Inform 2019; 95:103208. 31078660 10.1016/j.jbi.2019.103208 PMC7254481

