# Supplementary material for: Population Pharmacokinetics of Rifampicin in Plasma and Cerebrospinal Fluid in Adults With Tuberculosis Meningitis
Source: J Infect Dis. 2025 Apr 29;232(2):e234–41. doi: 10.1093/infdis/jiaf178 (PMC12349950; doi:10.1093/infdis/jiaf178)
Supplement: jiaf178_Supplementary_Data [file jiaf178_supplementary_data.docx]

# SUPPLEMENTARY MATERIAL: Population pharmacokinetics of rifampicin in plasma and cerebrospinal fluid in adults with tuberculosis meningitis

# Drug quantification

Rifampicin concentrations were measured with validated assays in the Division of Clinical Pharmacology laboratory at the University of Cape Town. The assays met FDA validation criteria. The total plasma assay involved a protein precipitation extraction, followed by isocratic liquid chromatographic separation, and mass spectrometry detection. The calibration range was 0.117 to 30 µg/mL. Plasma was separated using ultracentrifugation, and the plasma protein-free fraction was analyzed using solid-phase extraction and liquid chromatography with mass spectrometry detection. The calibration range was 0.0600 to 5.00 µg/mL. The CSF assay required protein precipitation and gradient liquid chromatography with mass spectrometry detection. The calibration range was 0.005 to 2.5 µg/mL.

# Imputation of missing covariates

Missing heights were imputed using multiple linear regression as suggested by Johansson and Karlsson [1] since it was missing in 60% of the participants. In the first step, participant characteristics, namely sex, weight, and height from a study in a similar population [2] were used to develop a multiple linear regression model for height versus weight by sex and accounting for residual variability in heights. Secondly, this multiple linear regression model was used to estimate the missing heights in NONMEM using a random effect model as shown in the equation below:

$$Ht_{i}=\beta+\alpha.Wt_{i}.e^{\eta_{i}}$$

Where $Ht_{i}$ is the individual height in meters and $Wt_{i}$ is the individual weight in kilograms. $\beta$ and $\alpha$are the model mean intercept and slope respectively. $\eta_{i}$ is the random effect accounting for the individual difference from the mean values. The $\eta_{i}$ values are assumed to be normally distributed with mean zero and variance $\omega^{2}$.The values of $\beta$ and $\alpha$ are 1.51 and 0.00133 for females and 1.53 and 0.00281 for males respectively. the values of $\omega_{2}$ were 0.00215 and 0.00170 for females and male respectively. NONMEM implementation can be found in the NONMEM code provided.

# Pharmacokinetic Modelling

## a) Nonlinear clearance

For the plasma model, the nonlinearity in clearance observed at higher doses was accounted for by a concentration-dependent $CL$ described by the following equation:

$$V_{max}= {CL}_{max}\cdot K_{m}$$

where $V_{max}$ is the maximal elimination rate in mg/h, ${CL}_{max}$ is the maximal clearance in L/h apparent with rifampicin plasma concentration ($C_{plasma}$) approximating 0, while $K_{m}$ is the $C_{plasma}$ in mg/L at which the elimination is half of $V_{max}$.

## b) Effect compartment modelling for CSF

The CSF concentrations were modelled as dependent on plasma concentrations using an effect compartment, as previously proposed and implemented by Sheiner et al. [3] and Savic et al [4]. Effect compartments are assumed to have a negligible volume compared to the central compartment, with negligible drug transfer between the two compartments. The following differential equation summarises the kinetics of the effect compartment:

$\frac{{dC}_{CSF}}{dt}=k_{plasma-CSF}\cdot\left( PPC\cdot C_{plasma}-C_{CSF} \right)$,

where $k_{plasma-CSF}$ is the first-order equilibration rate constant of the drug between the central compartment (i.e., plasma) and the effect compartment (i.e., CSF), $PPC$ is the pseudo-partition coefficient, $C_{plasma}$ and $C_{CSF}$ are the drug concentration at the time $t$ in plasma or CSF, respectively.

Between-subject, between-visit, and between-occasion variabilities were tested for the different plasma and CSF parameters. Each PK sampling day (day 3 and day 28) was considered a separate visit. Each dose and its following samples were considered a separate occasion, therefore the dose before the sampling visit along with the predose concentration were treated as a separate occasion from the dose administered during the PK visit and the following concentrations.

Residual unexplained variability was described using a combined proportional and additive error model, with the additive error for all samples set to be at least 20% of the LLOQ. Concentrations below the lower limit of quantification (BLQ) were censored according to Beal’s M6 method, in which the last censored value in a series during the absorption phase and the first censored value in a series in the terminal phase was replaced with LLOQ/2 and the other censored values in a series were discarded [2]. To account for the larger level of uncertainty in the imputed censored values, their additive error was inflated by LLOQ/2. Also, the M3 method was tested as there was a large fraction of BLQ values in the CSF but it did not result in a meaningful difference in the parameter estimates. On the other hand, it caused longer run times and, most importantly, less stable final parameter estimates. For this reason, we proceeded with the M6 method [2].

The process of model development and covariate inclusion was guided by physiological plausibility, model fit diagnostics, and the drop in the objective function value (OFV). The likelihood ratio test for the drop in OFV was used to compare nested models, assumed to be approximately χ2 distributed with n degrees of freedom, where n is the number of additional estimated parameters. A p-value of 0.05 was generally used for inclusion and 0.01 for retention. Model performance was evaluated through visual predictive checks (VPC). The VPC for the final model stratified into plasma and CSF concentrations is shown in **Figure S1**. Final parameters precision (95% confidence intervals) was obtained by sampling importance resampling (SIR) [5].

The model was developed using NONMEM^®^ 7.5 with first-order conditional estimation with eta-epsilon interaction (FOCE-I). Pirana 3.0.0 software was used for model management; Perl-speaks-NONMEM^®^ (PsN) 5.2.6 and R 4.0.4 via RStudio were used for post-processing NONMEM^®^ results and generating figures [6].

# Figures

**
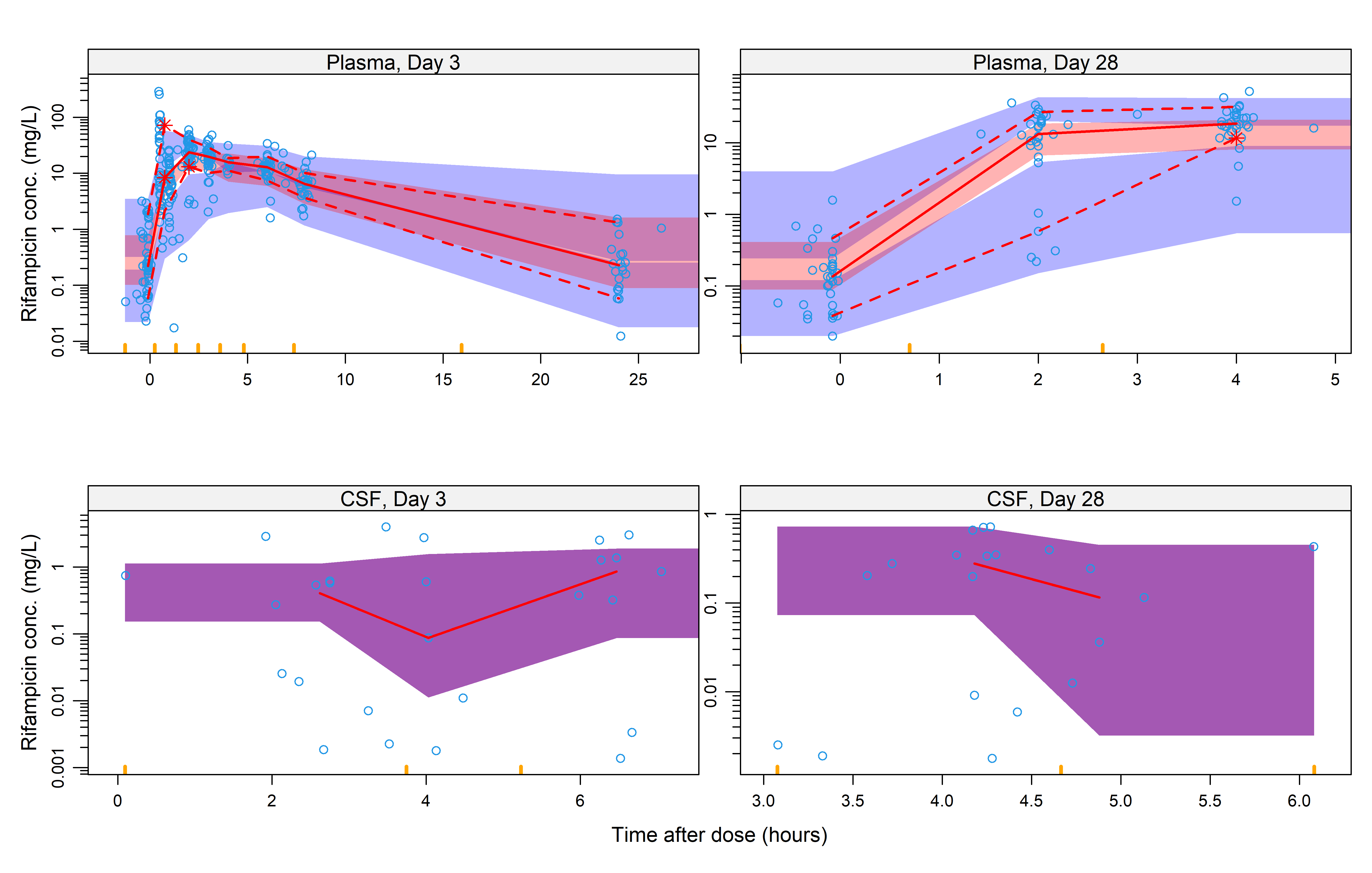
**

**Figure S1:** Prediction-corrected visual predictive check (VPC) (n=500) showing drug concentration versus time after dose for the final models stratified by matrix (plasma or CSF) and visit (day 3 or 28), dose. The dots are the original observations; the solid line is the median and the dashed lines are the 10^th^ and 90^th^ percentiles of the observed data; the shaded areas are the 90% confidence intervals of the same percentiles as simulated by the model. A suitably fitting model will have most of the observed percentiles within the simulated confidence intervals.

**
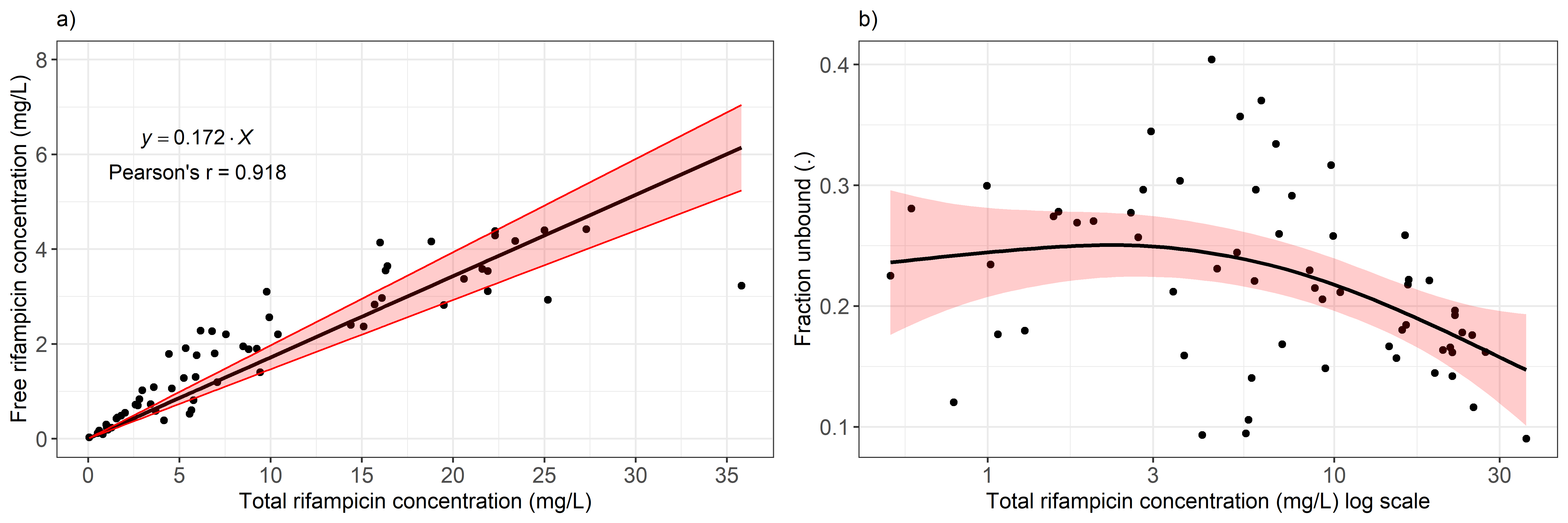
**

**Figure S2:** Binding plots for plasma protein binding of rifampicin a) Free versus total concentration (mg/L) in the same sample. The slope of the regression equation represents the unbound fraction and was estimated using generalised Deming regression with constant error assumption [7,8]. b) LOESS regression of fraction unbound versus total rifampicin concentrations (mg/L). There was no apparent trend between the two variables.

# NONMEM control file

;Model Description: RIF Final Model LASER-TBM Study

;Settings for the memory of NONMEM

$SIZES PD=-1000 LVR=-150 LTH=-200 MAXFCN=10000000 LNP4=-150000

DIMTMP=1000

$PROBLEM RIF_MODEL_LASER-TBM

$INPUT ID ....

$DATA data_rif_nm_2023-11-26.csv IGNORE=@ IGNORE(DVID.EQ.2)

IGNORE(DVID.EQ.3) IGNORE(DVID.EQ.4) IGNORE(FLAG_ENZ.GT.0)

IGNORE(ID.EQ.4019)

$SUBROUTINE ADVAN14 TRANS1 TOL=9 ATOL=9

$ABBREVIATED DERIV2=NO

$MODEL NCOMPS=7 ; NUMBER OF COMPARTMENTS

COMP=(ABS) ;1 GUT CMT

COMP=(CENTRAL) ;2 CENTRAL CMT

COMP=(LIVER) ;3 LIVER CMT

COMP=(PERI) ;4 PERI CMT

COMP=(CSF) ; 5 CSF CMT

COMP=(AUC_P) ; 6 AUC plasma

COMP=(AUC_CSF) ; 7 AUC CSF

;Using Priors------------------------------------------------

;Sim_start

$PRIOR NWPRI NPEXP=1 PLEV=0.9999

;Sim_end

;initialization-of-theta(S)-from the previous run----------

$THETA (0,1.09075,100) ; 1 LOGKM (mg/L) (exp)

$THETA (0,27.3473,125) ; 2 V (L)

$THETA (0,0.934124,1) ; 3 BIO_ORAL(.)

$THETA (0,0.485942,3) ; 4 KA (1/h)

$THETA (0,0.634202,3) ; 5 MTT (h)

$THETA (0,0.251954,1) ; 6 PROP (%)

;$THETA (0,1.63193E-05,10) ; 7 ADD (mg/L)

$THETA 0 FIX ; 7 ADD (mg/L)

$THETA 1 FIX ; 8 VH

$THETA 90 FIX ; 9 QH

$THETA 1 FIX ; 10 BIO_IV()

$THETA (0,10.9971,1000) ; 11 Q

$THETA (0,31.5279,1000) ; 12 VP

$THETA (0,19,100) FIX ; 13 NN (.)

;----------------------------

$THETA (0,46.1064,1000) ; 14 CL_Day3_High

$THETA (0,33.1219,1000) ; 15 CL_Day3_Std

$THETA (0,70.2333,1000) ; 16 CL_Day28_High

$THETA (0,41.411,1000) ; 17 CL_Day28_Std

;----------------------------

$THETA (0,3.19641,10) ; 18 EQHR (h) --> KE0

$THETA (0,0.059288,1) ; 19 PPC

$THETA (0,0.984216,1) ; 20 PROP_CSF []

$THETA (0,0.0206258,10) ; 21 ADD_CSF [mg/L]

;----------------------------

;THETA Priors (Reference values from reference model of the THETAs)

;Sim_start

$THETAP 1.21 FIX ; LOGKM

;Sim_end

;Sim_start

$THETAPV BLOCK(1) FIX

0.1 ; LOGKM

;Sim_end

$OMEGA BLOCK(1)

0.0642298 ; 1 BSVCL

$OMEGA BLOCK(1)

0.0295196 ; 2 BSVV

$OMEGA BLOCK(1) FIX

0 ; 3 BSVBIO

$OMEGA BLOCK(1)

0.0329715 ; 4-8 BOVBIO

$OMEGA BLOCK(1) SAME

$OMEGA BLOCK(1) SAME

$OMEGA BLOCK(1) SAME

$OMEGA BLOCK(1) SAME

$OMEGA BLOCK(1)

0.609918 ; 9-13 BOVKA

$OMEGA BLOCK(1) SAME

$OMEGA BLOCK(1) SAME

$OMEGA BLOCK(1) SAME

$OMEGA BLOCK(1) SAME

$OMEGA BLOCK(1)

1.23318 ; 14-18 BOVMTT

$OMEGA BLOCK(1) SAME

$OMEGA BLOCK(1) SAME

$OMEGA BLOCK(1) SAME

$OMEGA BLOCK(1) SAME

$OMEGA BLOCK(1) FIX

0 ; 19-20 BVVCL

$OMEGA BLOCK(1) SAME

$OMEGA BLOCK(1)

0.0288515 ; 21 BSV D2

$OMEGA 0.00215 FIX ; 22 Variance HTfemale

$OMEGA 0.00170 FIX ; 23 Variance HTmale

$OMEGA BLOCK(1) FIX

0 ; 24-25 BVVPPC

$OMEGA BLOCK(1) SAME

$OMEGA BLOCK(1) FIX

0 ; 26 BSVPPC

$SIGMA 1 FIX

$SIGMA 1 FIX

$PK

;-------Typical values of covariates

TVWT = 60 ;median wt from the data set

TVFAT = 14

TVFFM = 46

;---------Allometric scaling and covariates

; ALLMCL_FFM = (FFM_IMP/TVFFM)**0.75

; ALLMV_FFM = (FFM_IMP/TVFFM)

;- Imputation of HT and FFM all individuals -

IMP_HTM = ((0.00133*WT) + 1.51)*EXP(ETA(22)) ;for females

IF (SEXF.EQ.0) IMP_HTM = ((0.00281*WT) + 1.53)*EXP(ETA(23)) ;for males

IMP_FFM = (37.99 * (IMP_HTM**2) * WT) / (35.98 * (IMP_HTM**2) + WT); for females

IF (SEXF.EQ.0) IMP_FFM = (42.92 * (IMP_HTM**2) * WT) / (30.93 * (IMP_HTM**2) + WT);for males

IF(FFM.NE.-99) ALLMCL_FFM = (FFM/TVFFM)**0.75

IF(FFM.EQ.-99) ALLMCL_FFM = (IMP_FFM/TVFFM)**0.75

IF(FFM.NE.-99) ALLMV_FFM = (FFM/TVFFM)

IF(FFM.EQ.-99) ALLMV_FFM = (IMP_FFM/TVFFM)

;Hepatic allometry

IF(FFM.NE.-99) ALLMCL_H_FFM = (FFM/56.1)**0.75

IF(FFM.EQ.-99) ALLMCL_H_FFM = (IMP_FFM/56.1)**0.75

IF(FFM.NE.-99) ALLMV_H_FFM = (FFM/56.1)

IF(FFM.EQ.-99) ALLMV_H_FFM = (IMP_FFM/56.1)

;PARAMTERS

TVLOGKM = THETA(1) ; LOGKM

TVV = THETA(2)*ALLMV_FFM

TVBIO_ORAL = THETA(3)

TVKA = THETA(4)

TVMTT = THETA(5)

TVBIO_IV = THETA(10)

TVCL = THETA(14)*ALLMCL_FFM

IF (RIFHIGH.EQ.0.AND.PK_VISIT_2.EQ.3) TVCL = THETA(15)*ALLMCL_FFM

IF (RIFHIGH.EQ.1.AND.PK_VISIT_2.EQ.28) TVCL = THETA(16)*ALLMCL_FFM

IF (RIFHIGH.EQ.0.AND.PK_VISIT_2.EQ.28) TVCL = THETA(17)*ALLMCL_FFM

TVVH = THETA(8)*ALLMV_H_FFM

TVQH = THETA(9)*ALLMCL_H_FFM

TVQ = THETA(11)*ALLMCL_FFM

TVV2 = THETA(12)*ALLMV_FFM

TVNN = THETA(13)

TVEQHR = THETA(18)

TVPPC = THETA(19)

;Defining ETAs

BSVCL = ETA(1)

BSVV = ETA(2)

BSVBIO = ETA(3)

;Defining Between OCC variability;

BOVBIO = 0

BOVKA = 0

BOVMTT = 0

IF (OCC==1) THEN ;PREDOSE PK_VISIT=3

BOVBIO = ETA(4)

BOVKA = ETA(9)

BOVMTT = ETA(14)

;BOVCL = ETA(19)

ENDIF

IF (OCC==2) THEN

BOVBIO = ETA(5)

BOVKA = ETA(10)

BOVMTT = ETA(15)

;BOVCL = ETA(19)

ENDIF

IF (OCC==3) THEN

BOVBIO = ETA(6)

BOVKA = ETA(11)

BOVMTT = ETA(16)

;BOVCL = ETA(19)

ENDIF

IF (OCC==4) THEN

BOVBIO = ETA(7)

BOVKA = ETA(12)

BOVMTT = ETA(17)

;BOVCL = ETA(20)

ENDIF

IF (OCC==5) THEN

BOVBIO = ETA(8)

BOVKA = ETA(13)

BOVMTT = ETA(18)

;BOVCL = ETA(20)

ENDIF

BVVCL = 0

IF (PK_VISIT==3) BVVCL=ETA(19)

IF (PK_VISIT==28) BVVCL=ETA(20)

BSVD2 = ETA(21)

BVVPPC = 0

IF (PK_VISIT==3) BVVPPC=ETA(24)

IF (PK_VISIT==28) BVVPPC=ETA(25)

BSVPPC = ETA(26)

;----------------------------------------------------------

CL = (TVCL)*EXP(BSVCL+BVVCL)

V = (TVV)*EXP(BSVV)

KA = (TVKA)*EXP(BOVKA)

MTT = (TVMTT)*EXP(BOVMTT)

NN = TVNN

D2 = DUR*EXP(BSVD2)

BIO_ORAL = (TVBIO_ORAL)*EXP(BSVBIO+BOVBIO)

BIO_IV = TVBIO_IV

VH=TVVH

QH=TVQH

LOGKM = TVLOGKM;*EXP(BSVKM)

VMAX = CL*EXP(LOGKM) ; max enzymatic rate

Q = TVQ

V2 = TVV2

EQHR = TVEQHR

KE0 = LOG(2)/EQHR

PPC = TVPPC*EXP(BVVPPC+BSVPPC)

K24 = Q/V

K42 = Q/V2

F2 = 0

IF(RIFIV.EQ.1) F2=BIO_IV

;Transit code

F1=0 ; I need to set bioavailability in compartment 1 to 0 for this implementation of the transit compartment absorption

KTR = (NN+1)/MTT ; The number of actual transit compartments is NN+1, so this number can never be 0

IF (NEWIND/=2.OR.EVID>=3.AND.CMT.EQ.1) THEN ; new individual, or reset event

; The values read here will be stored in TDOS and PD in this very PK call.

TNXD=TIME ; Time of the dose

PNXD=AMT ; Amount. If it's zero, the DE is deactivated.

TIMEDOSE = TIME

AMOUNTDOSE = AMT

ENDIF

TDOS=TNXD ; This will either save here the temporary values if it's a new individual...

PD=PNXD ; ...or the values which were read one record ahead during the execution of the previous record.

IF(AMT>0.AND.CMT.EQ.1) THEN ; This reads one record ahead and stores the data to be used when running the following record

; IF(AMT.GT.0.AND.ALAG1.EQ.0) THEN ; Use this INSTEAD if there is ALAG, as it will also checks if the ALAG is not 0. Note that you normally do not want to include both ALAG and transit, this is a very exceptional case

TNXD=TIME

PNXD=AMT

ENDIF

PIZZA = LOG(BIO_ORAL*PD*KTR + 1E-12) - GAMLN(NN+1) ; without +0.00001, it won't work with ETAs in bioavailability

A_0(1) = 1E-12 ;ABS

A_0(2) = 1E-12 ;CENT

A_0(3) = 1E-12 ;LIV

A_0(4) = 1E-12 ;PERI

A_0(5) = 1E-12 ;CSF

;----------------------------------------------------------

**$DES**

;Transit code

TEMPO = T-TDOS ; this is time after dose for the transit, it should always be >= 0

KTT = 0

TRANSIT = 0

IF(PD.GT.0.AND.TEMPO.GT.0) THEN ; This happens only id PD>0, so only if a dose has been detected

KTT = KTR*(TEMPO)

TRANSIT = EXP(PIZZA+NN*LOG(KTT)-KTT)

ENDIF

;Saturable CL (Michaelis-Menten)

CH = A(3)/VH ;Conc. in liver

SAT_CL=0

IF (CH>0) SAT_CL = VMAX / (CH + EXP(LOGKM))

EH = (SAT_CL)/((SAT_CL)+QH) ; fraction undergoing first pass extraction

FH = 1 - EH ;fraction available after 1st pass to go to systemic circulation

K30=(QH*EH/VH)

K32= (QH*FH/VH) ;from liver to plasma

K23=(QH/V) ;frm plasma to liver

DADT(1) = TRANSIT-KA*A(1) ;gut

DADT(2) = K32*A(3)-K23*A(2) - K24*A(2) + K42*A(4);plasma

DADT(3) = KA*A(1)-K32*A(3)+K23*A(2)-K30*A(3) ;liver

DADT(4) = K24*A(2)-K42*A(4) ; 2nd cmt

CP_DES = A(2)/V

DADT(5) = KE0*(PPC*CP_DES - A(5)) ;CSF

DADT(6) = CP_DES

DADT(7) = A(5)

;----------------------------------------------------------

**$ERROR**

; DEFINE LLOQ VALUE

; LLOQ could be study-specific, e.g if you have data from different labs in your analysis

LLOQ_P = 0.117 ; DEFINE YOUR OWN LLOQ HERE

; DEFINE censoring threshold (CENS_THR)

; Generally the same as LLOQ, but not if the LLOQ data was released by the lab.

; If censoring threshold is not explicitly indicated, we can generally assume it to be the limit of detection (LOD).

; The signal-to-noise ratio is generally assumed to be 10 at the LLOQ, and 3 at the LOD

; https://en.wikipedia.org/wiki/Detection_limit;

; Keizer RJ, Jansen RS, Rosing H, Thijssen B, Beijnen JH, Schellens JHM, Huitema ADR.

; Incorporation of concentration data below the limit of quantification in population pharmacokinetic analyses.

; Pharmacol Res Perspect [Internet]. 2015 Mar;3(2):e00131. Available from: http://doi.wiley.com/10.1002/prp2.131

CENS_THR_P = LLOQ_P

CP = A(2)/V

IPRED_P = CP

PROP_P = IPRED_P*THETA(6)

ADD_P = THETA(7)+(CENS_THR_P*0.2)

; ADD is defined as 20% of LLOQ + THETA(.)

IF (ICALL/=4.AND.CENS==1.AND.DVID==1) THEN

ADD_P = ADD_P +(CENS_THR_P*0.5)

ENDIF

W_P = SQRT((ADD_P)**2 + (PROP_P)**2)

ERROR_P = W_P * ERR(1)

NO_FIT = 0

CENS_THR_VPC_P = CENS_THR_P

IMPUTED_CENS_VPC_P = CENS_THR_P/2

; For CENS==2 (i.e. the trailing CENSORED values in a series that were imputed to CENS_THR/2), we don't want these to influence the fit,

; we only want them for simulation-based diagnostics such as the VPC.

; So we define a separate error structure for these points. It has no proportional component

; (PROP = 0, as we would not want these points to affect our estimate of proportional error)

; and a FIXED and HUGE additive component (ADD = 1000000000, large with respect to the readings of concentration),

; so that the values do not affect the fit. It's also a good idea to repeat the diagnostic plots without the CENS=2 points

IF (ICALL/=4.AND.CENS==2.AND.DVID==1) THEN

PROP_P = 0

ADD_P = 10000000000

NO_FIT = 1

ENDIF

;Repeat for CSF observations------------------------------------------

LLOQ_E = 0.005

CENS_THR_E = LLOQ_E

CE = A(5)

IPRED_E = CE

PROP_E = IPRED_E*THETA(20)

ADD_E = THETA(21)+(CENS_THR_E*0.5)

IF (ICALL/=4.AND.CENS==1.AND.DVID==5) THEN

ADD_E = ADD_E +(CENS_THR_E*0.5)

ENDIF

W_E = SQRT((ADD_E)**2 + (PROP_E)**2)

ERROR_E = W_E * ERR(2)

NO_FIT = 0

CENS_THR_VPC_E = CENS_THR_E

IMPUTED_CENS_VPC_E = CENS_THR_E/2

IF (ICALL/=4.AND.CENS==2.AND.DVID==5) THEN

PROP_E = 0

ADD_E = 10000000000

NO_FIT = 1

ENDIF

;---------------------------------------------------------------------

;Redefine IPRED & weighting

IPRED = IPRED_P

W = W_P

ERROR_TERM = ERROR_P

CENS_THR_VPC = CENS_THR_VPC_P

IMPUTED_CENS_VPC = IMPUTED_CENS_VPC_P

IF (DVID==5) THEN

IPRED = IPRED_E

W = W_E

ERROR_TERM = ERROR_E

CENS_THR_VPC = CENS_THR_VPC_E

IMPUTED_CENS_VPC = IMPUTED_CENS_VPC_E

ENDIF

; Protective code

IF (W.LE.0.000001) W=0.000001

IRES=DV-IPRED

IWRES=IRES/W

Y = IPRED + ERROR_TERM

; To prevent simulation (ICALL==4) of negative values. It set a positive lower bound for Y, so that VPCs in the log-scale can be plotted

IF (ICALL==4.AND.Y<=CENS_THR_VPC) Y = IMPUTED_CENS_VPC

; To calculate time after dose.

IF(AMT.GT.0) THEN

TIMEDOSE = TIME

AMOUNTDOSE = AMT

ENDIF

TAD = TIME-TIMEDOSE

;;Retrieve amount in each compartment

A_GUT = A(1)

A_CENT = A(2)

A_LIV = A(3)

A_PERI = A(4)

A_CSF = A(5)

AUC_P = A(6)

AUC_CSF = A(7)

**$ESTIMATION** METHOD=1 INTER MAXEVAL=9999 PRINT=1 NOABORT NSIG=3 SIGL=6

            NONINFETA=1 ETASTYPE=1

# References

1. Johansson ÅM, Karlsson MO. Multiple imputation of missing covariates in NONMEM and evaluation of the method’s sensitivity to η-shrinkage. AAPS J. **2013**; 15(4):1035–1042.

2. Brust JCM, Gandhi NR, Wasserman S, et al. Effectiveness and Cardiac Safety of Bedaquiline-Based Therapy for Drug-Resistant Tuberculosis: A Prospective Cohort Study. Clin Infect Dis An Off Publ Infect Dis Soc Am [Internet]. Oxford University Press; **2021** [cited 2023 Aug 28]; 73(11):2083. Available from: /pmc/articles/PMC8664482/

3. Sheiner LB, Stanski DR, Vozeh S, Miller RD, Ham JS, Francisco C. Simultaneous modeling of pharmacokinetics and pharmacodynamics: Application to d-tubocurarine. 1979.

4. Savic R, Ruslami R, Hibma J, et al. Pediatric tuberculous meningitis: Model-based approach to determining optimal doses of the anti-tuberculosis drugs rifampin and levofloxacin for children. Clin Pharmacol Ther [Internet]. **2015**; 98(6):622–629. Available from: https://onlinelibrary.wiley.com/doi/10.1002/cpt.202

5. Dosne AG, Bergstrand M, Harling K, Karlsson MO. Improving the estimation of parameter uncertainty distributions in nonlinear mixed effects models using sampling importance resampling. J Pharmacokinet Pharmacodyn. **2016**; 43(6):583–596.

6. Keizer RJ, Karlsson MO, Hooker A. Modeling and simulation workbench for NONMEM: Tutorial on Pirana, PsN, and Xpose. CPT Pharmacometrics Syst Pharmacol. 2013/07/10. Department of Pharmaceutical Biosciences, Pharmacometrics research Group, Uppsala University, Uppsala, Sweden.; **2013**; 2(6):1–9.

7. Deming WE. Statistical adjustment of data. Stat. Adjust. data. Oxford, England: Wiley; 1943.

8. Linnet K. Performance of Deming regression analysis in case of misspecified analytical error ratio in method comparison studies. Clin Chem [Internet]. **1998**; 44(5):1024–31. Available from: http://www.ncbi.nlm.nih.gov/pubmed/9590376
